# Author Correction: Replay without sharp wave ripples in a spatial memory task

**DOI:** 10.1038/s41467-026-72252-8

**Published:** 2026-04-17

**Authors:** John Widloski, David J. Foster

**Affiliations:** 1 Allen Institute for Neural Dynamics, Seattle, WA USA; 2 Helen Wills Neuroscience Institute and Department of Psychology, University of California, Berkeley, CA USA

**Keywords:** Spatial memory, Hippocampus

Correction to: *Nature Communications* 10.1038/s41467-025-65181-5, published online 21 November 2025

In the version of the article initially published, refs. 6, 7, 17, 23, 25, 29, 21, 42, 46, 49, 57, 58, 63, 64, 66 and 67 were incorrect and have now been amended in the HTML and PDF versions of the article.

