# Supplementary material for: Replay without sharp wave ripples in a spatial memory task
Source: Nat Commun. 2025 Nov 21;16:10287. doi: 10.1038/s41467-025-65181-5 (PMC12639102; doi:10.1038/s41467-025-65181-5)
Supplement: Supplementary file 1 — Supplementary Information [file 41467_2025_65181_MOESM1_ESM.pdf]

SUPPLEMENTAL INFORMATION

Replay without sharp wave ripples in a spatial memory task.

John Widloski & David Foster

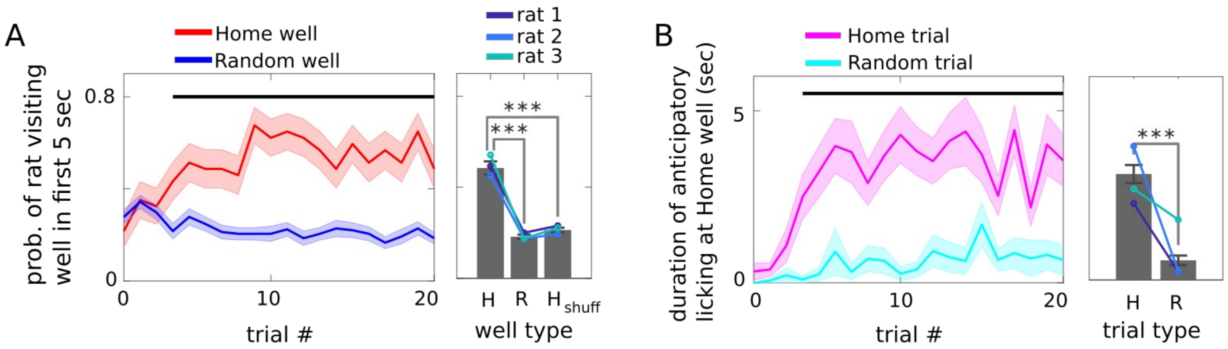

Supplemental Fig. 1. (**A**) Probability of the rat visiting the Home well (H) versus a Random well (R) within the first 5 s of the Home trial, as a function of trial number (left) or averaged across trials (right). Horizontal black line: $p < 0.05$. H_{shuff} is calculated the same as H except that the Home well ID was selected randomly. Data presented as mean +- SEM. Colored lines indicate means for individual rats. Wilcoxon signed rank test (two-sided); ***$p<0.001$. (**B**) Duration of anticipatory licking (see Methods) at the Home well for Home (H) versus Random (R) trials as a function of trial number (left) or averaged across trials (right). Horizontal black line, $p < 0.05$.

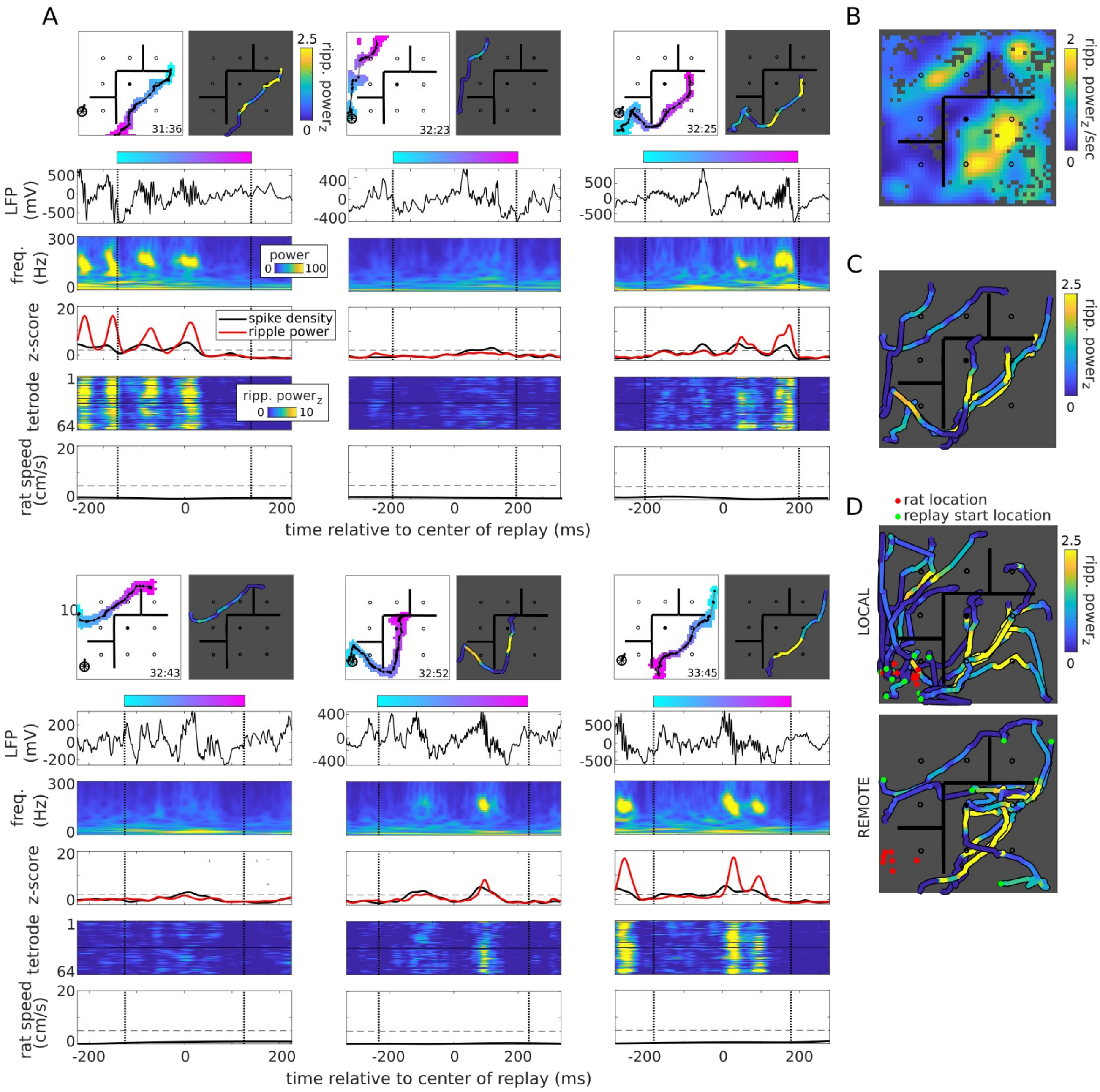

Supplemental Fig. 2. (**A**) 6 replays from rat 1, session 75, taken from the the same stopping period and depicted as in Figure 1. For each replay, the colored blob in the upper left panel depicts the posterior probability of the replay thresholded at 0.01 and color-coded according to elapsed time within the replay. Solid black line: replay center-of-mass. Time within session is shown at the upper left (min:sec). Black circles and straight lines are the locations of reward and transparent barriers, respectively. Filled circle is the home well. The panel at upper right shows the ripple power (averaged across tetrodes) superimposed on the replay center-of-mass. Second row: Raw LFP trace from single electrode as a function of time within the

replay. Black vertical dashed lines demarcate start and end of replay. Third row: Spike density summed across all recorded cells (black) and ripple power (red). Dashed horizontal line indicates z-score of 3. Fourth row: Ripple power across tetrodes. Tetrodes 1-32 (33-64) targeted the left (right) hemisphere. Fifth row: Rat speed. Dashed horizontal line indicates rat speed of 5 cm/s. (**B**) Ripple field for the same session. (**C**) Same 6 replays depicted in (**A**), with ripple power superimposed over the center-of-mass. Note the peaks in ripple power correspond to the ripple field location in (**B**). (**D**) Replays from the same stopping period, including the replays in (**A**), divided into “local” and “remote” replays, depending on if the replay starting location is less than or greater than 20 cm from the rat. Red dots indicate position of the rat during each replay. Green dots indicate replay start position.

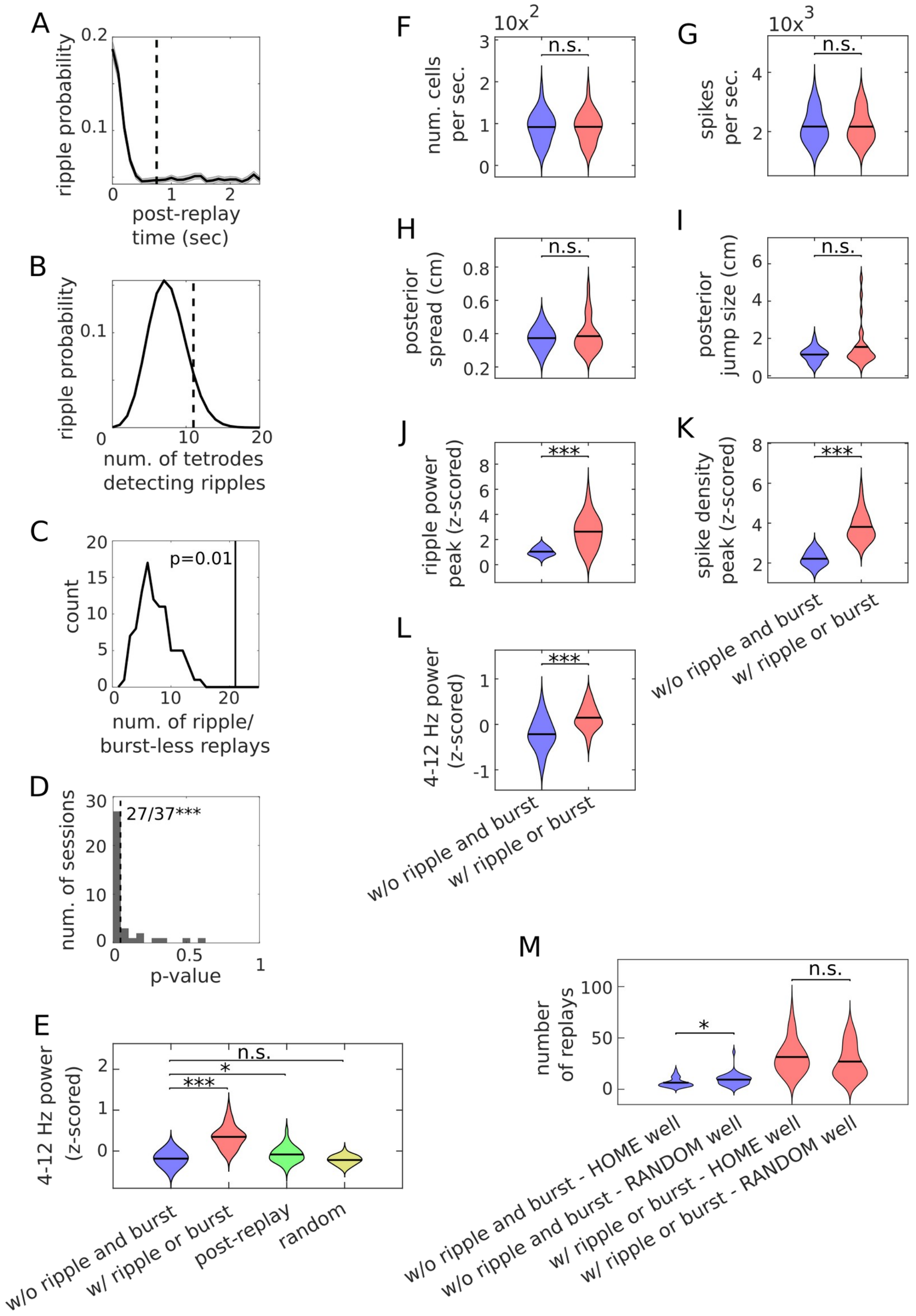

Supplemental Fig. 3. (**A**) Probability of detecting a ripple as a function of time since the end of replay, averaged across sessions. To compute this, for each replay, ripples were detected (from ripple power averaged across across tetrodes) within replay-length windows shifted in time from end of the replay. This was then averaged across replays and across sessions (n =

37 sessions). Dashed vertical line indicates a shift of 0.75 sec. Data depicted as mean +- SEM. (**B**) Probability of detecting a ripple computed from the binomial distribution with 64 tetrodes and a probability of ripple detection per tetrode of 12%. Dashed vertical lines indicates 95th percentile of distribution, which occurs at 11 tetrodes. The "success rate" of 12% was computed as follows: for tetrode and for each replay, select replay-length windows at random throughout the stopping period and determine whether a ripple occurs or not. Repeat this 100 times to compute ripple probability, then average this number across all replays, all tetrodes, and all sessions. (**C**) Distribution of the number of ripple/burst-less replays using shuffled place-cell IDs (100 shuffles; black line) compared to the original data set (vertical solid line), for rat 1, session 75. Replays from the shuffled place-cell decoding were found as before. The p-value is computed as the fraction of shuffles with ripple/burst-less replay counts larger than for the original data set (one-sided). (**D**) Distribution of p-values for significance of ripple/burst-less replay counts across sessions. Dashed vertical line is 0.05. Number of significant sessions indicated at upper right, with p-value computed from the Fisher combined probability test (p-value = 1.14×10^{-23}). (**E**) 4-12 power (z-scored) for ripple/burst-less replays (blue), replays with bursts or ripples (red), post-replay periods (0.75 sec after replay; green), and from replay-length windows sampled randomly throughout the stopping period (yellow), averaged across sessions (n = 37 sessions; two-sample t-tests, two-sided). P-values: w/o vs. w/ ripple/burst: 7.68×10^{-30}, w/o ripple/burst vs. post-replay: 2.06×10^{-27}, w/o ripple/burst vs. random: 3.6×10^{-46}. (**F-G**) Number of spikes per second (**F**) and number of active cells per second (**G**) for each replay averaged across sessions, after matching the joint distribution of these two measures across ripple/burst-less replays and replays with bursts or ripples within each session. This was achieved by constructing a two-dimensional histogram over the two measures for each replay type and downsampling events from both groups to retain only the overlapping portions of the distributions. (**H-L**) Mean posterior spread (**H**), jump size (**I**), peak ripple power (**J**), peak spike density (**K**), and 4-12 Hz power (**L**) for ripple/burst-less replays (blue) compared to replays with ripples or bursts (red) using the matched data set from (**F-G**), averaged across sessions (n = 37 sessions; two-sample t-tests, two-sided). P-values for (**F-L**): 0.95, 0.95, 0.77, 1.9×10^{-8}, 4.35×10^{-15}, 5.48×10^{-6}. (**M**) Total number of ripple/burst-less replays and replays without ripples or bursts for when the rat is at the Home well vs. a Random well, averaged across sessions. (n = 37 sessions; two-sample t-tests, two-sided, (*p<0.05, **p<0.01; ***p<0.001)

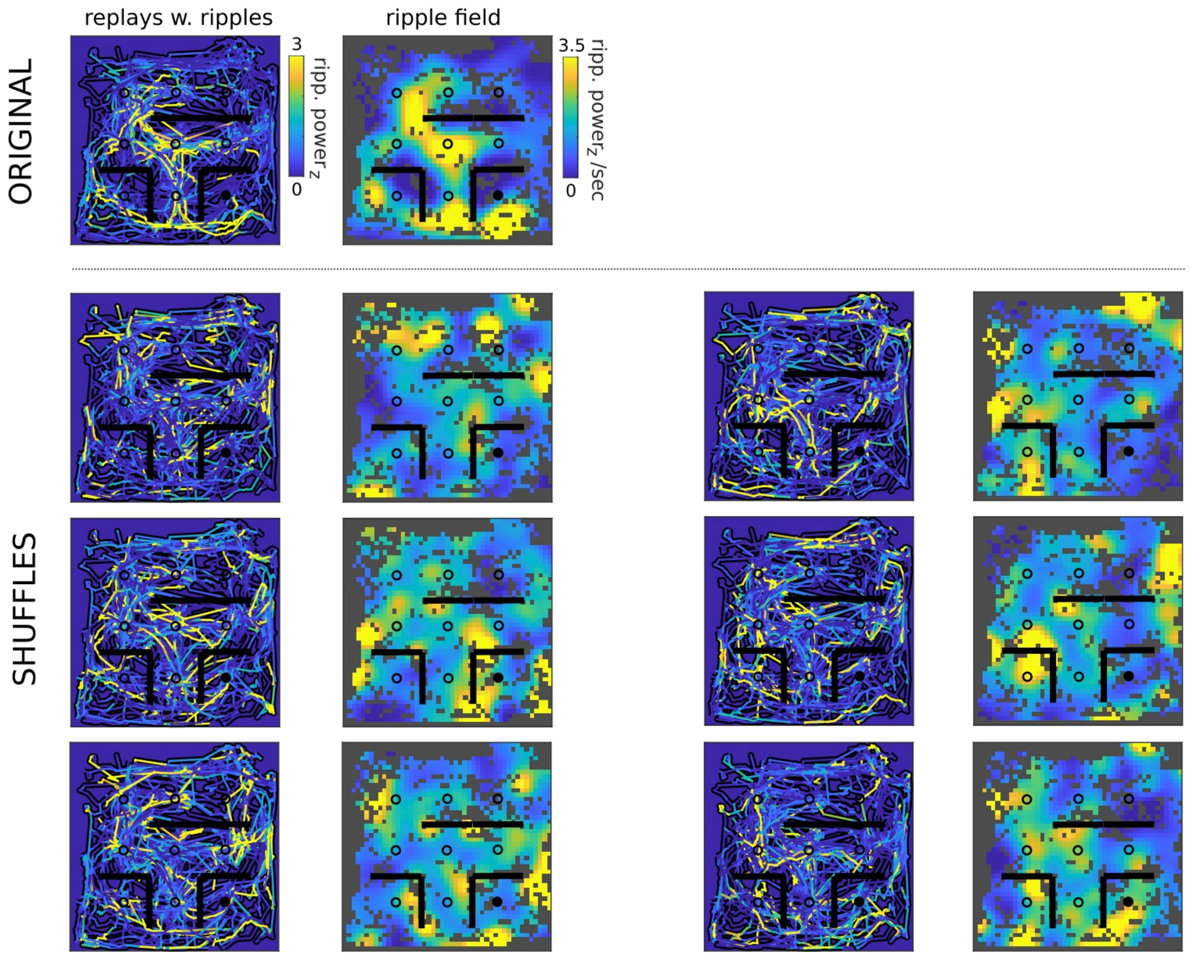

Supplemental Fig. 4. Top: Replays with ripple power superimposed (left) and corresponding ripple field (right) for session 76, rat 1. Bottom: 6 shuffle examples, where for each pair, ripple power has been circularly permuted across all replay events.

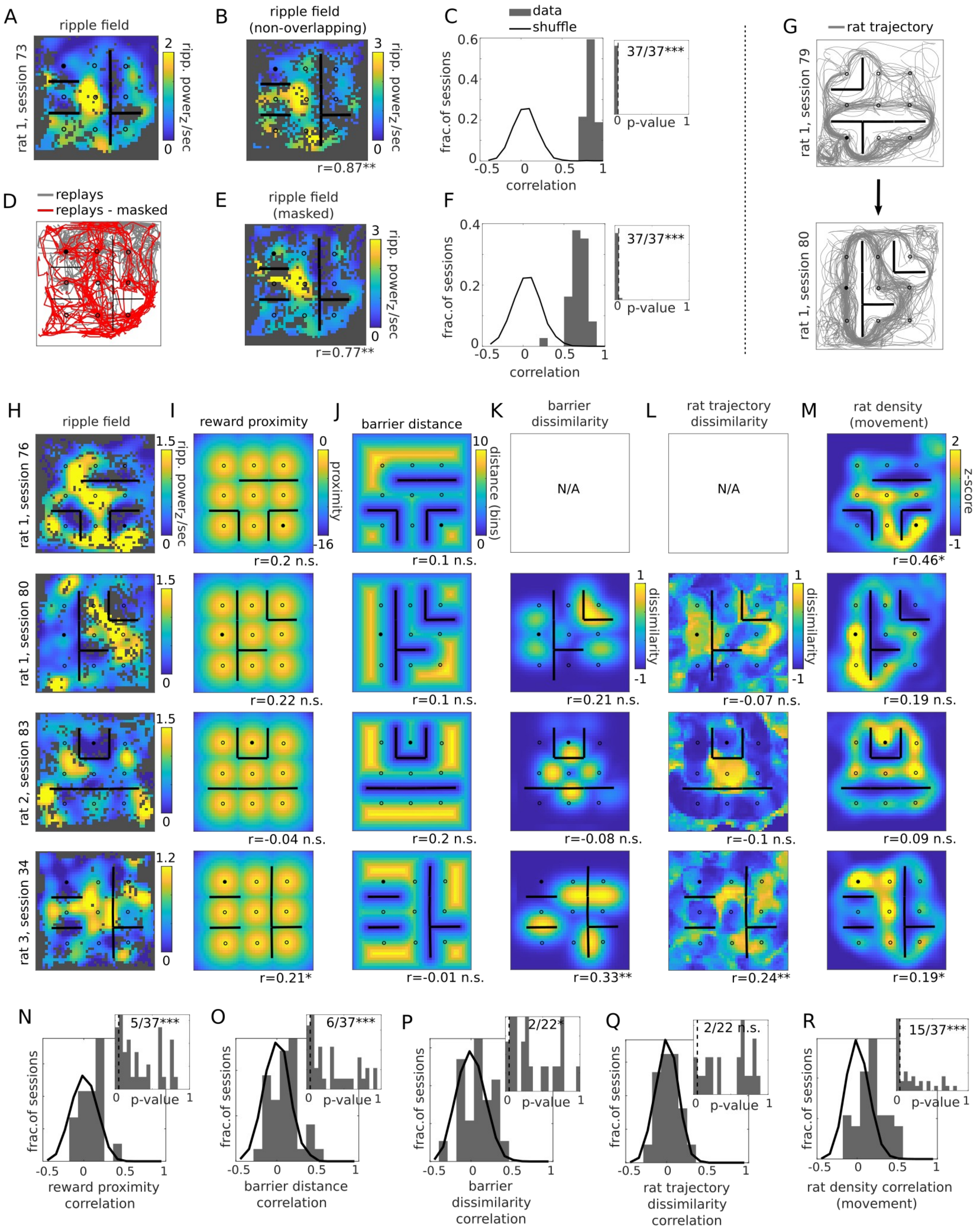
A
ripple field
rat 1, session 73
ripp. powerz/sec
B
ripple field (non-overlapping)
r=0.87**
C
data
shuffle
frac.of sessions
correlation
37/37***
p-value
D
replays
replays - masked
E
ripple field (masked)
r=0.77**
F
37/37***
G
rat trajectory
rat 1, session 79
rat 1, session 80
H
ripple field
I
reward proximity
proximity
J
barrier distance
distance (bins)
K
barrier dissimilarity
L
rat trajectory dissimilarity
M
rat density (movement)
z-score
rat 1, session 76
N/A
N/A
r=0.2 n.s.
r=0.1 n.s.
r=0.46*
rat 1, session 80
dissimilarity
r=0.22 n.s.
r=0.1 n.s.
r=0.21 n.s.
r=-0.07 n.s.
r=0.19 n.s.
rat 2, session 83
r=-0.04 n.s.
r=0.2 n.s.
r=-0.08 n.s.
r=-0.1 n.s.
r=0.09 n.s.
rat 3, session 34
r=0.21*
r=-0.01 n.s.
r=0.33**
r=0.24**
r=0.19*
N
5/37***
reward proximity correlation
O
6/37***
barrier distance correlation
P
2/22*
barrier dissimilarity correlation
Q
2/22 n.s.
rat trajectory dissimilarity correlation
R
15/37***
rat density correlation (movement)

Supplemental Fig. 5. (**A**) Ripple field from rat 1, session 73. (**B**) Ripple field from rat 1, session 73 computed using replays decoded using non-overlapping 20 msec bins. The spatial correlation with the original ripple field in (**A**) is listed below. Significance was assessed by circularly permuting ripple power 100 times to recompute the ripple field in (**A**) and measuring the fraction of shuffles with spatial correlation greater than the actual value. (**C**) Distribution of spatial correlations across sessions (n = 37 sessions) between ripple fields computed with and without non-overlapping bins. Session shuffles are shown as empty histogram. Inset: Distribution of p-values across sessions, with number of significant sessions indicated. Dashed vertical line is 0.05. Number of significant sessions indicated at upper right. Significance computed from Fisher combined probability test. (**D**) All replays from rat 1, session 73 (gray). The red lines depict the same replays, but with portions of each replay that are within 30 cm from the rat's location cut out ("masked"). (**E**) Ripple field from rat 1, session 73 computed using the "masked" replays in (**D**). The spatial correlation with the original ripple field in (**A**) is listed below, with significance computed as in (**B**). (**F**) Distribution of spatial correlations and p-values across sessions between ripple fields compute with and without "masking". (**G**) Rat trajectory during movement (speed > 10 cm/sec) for back to-back-sessions (related to panels K-L below). (**H**) Ripple fields across 4 sessions (same as Figure 2C). (**I-M**) Reward proximity (**I**); distance to nearest barrier (**J**); barrier dissimilarity (**K**) and rat trajectory dissimilarity (**L**) between current and previous sessions as depicted in (**B**) – see Methods; rat trajectory density during movement (rat speed > 10 cm/sec; **M**). Below each panel is the spatial correlation with the corresponding ripple field in (**H**). The blank panels in (**K-L**) for row 1 are because session 76 was the first session of the day. (**N-R**) Distribution of spatial correlations for the measures in (**I-M**) with the ripple fields, computed across sessions. Significance computed from Fisher combined probability test. P-values for (**N-R**): 9.27×10^{-4}, 2.66×10^{-4}, 0.01, 0.24, 2.18×10^{-10} (*p<0.05, ***p<0.01).

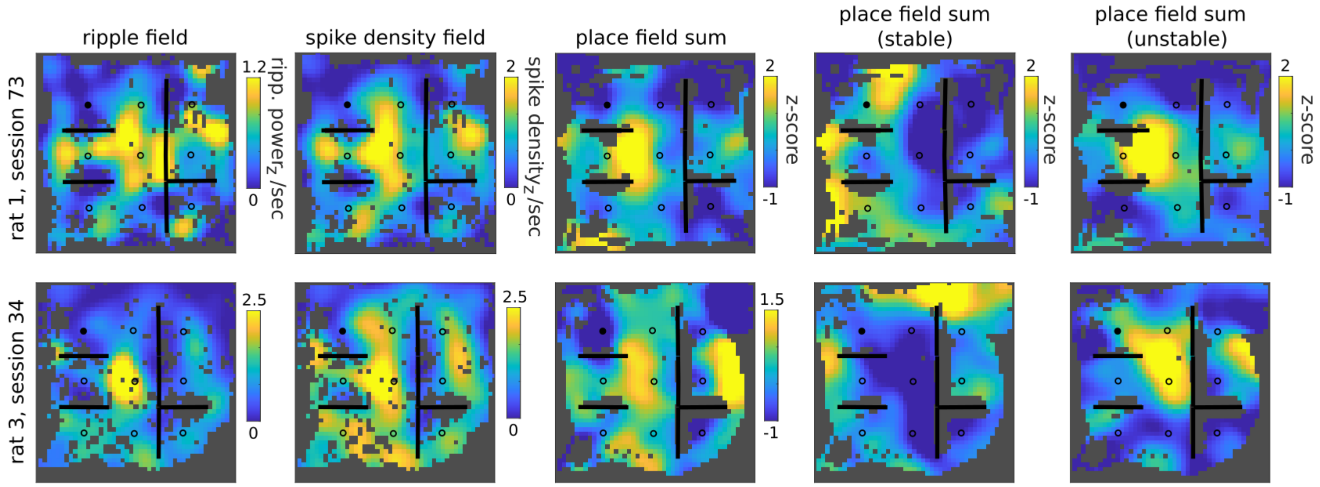

Supplemental Fig. 6. Comparison of ripple fields with summed stable and unstable place fields for 2 sessions (rows). From left to right: Ripple field, spike density field, place field sum, and place field sum for stable and unstable cells.
